# Identifying protective host gene expression signatures within the spleen during West Nile virus infection in the collaborative cross model

**DOI:** 10.1016/j.gdata.2016.10.006

**Published:** 2016-10-14

**Authors:** Richard Green, Courtney Wilkins, Sunil Thomas, Aimee Sekine, Renee C. Ireton, Martin T. Ferris, Duncan M. Hendrick, Kathleen Voss, Fernando Pardo-Manuel de Villena, Ralph Baric, Mark Heise, Michael Gale

**Affiliations:** a Department of Immunology, Center for Innate Immunity and Immune Disease (CIIID), University of Washington, Seattle, Washington, USA; b Department of Epidemiology, University of North Carolina at Chapel Hill, North Carolina, USA

**Keywords:** Collaborative cross, Flaviviruses, Nanostring, West Nile virus, Spleen

## Abstract

Flaviviruses are hematophagous arthropod-viruses that pose global challenges to human health. Like Zika virus, West Nile Virus (WNV) is a flavivirus for which no approved vaccine exists [1]. The role host genetics play in early detection and response to WNV still remains largely unexplained. In order to capture the impact of genetic variation on innate immune responses, we studied gene expression following WNV infection using the collaborative cross (CC). The CC is a mouse genetics resource composed of hundreds of independently bred, octo-parental recombinant inbred mouse lines [2]. To accurately capture the host immune gene expression signatures of West Nile infection, we used the nanostring platform to evaluate expression in spleen tissue isolated from CC mice infected with WNV over a time course of 4, 7, and 12 days' post-infection [3]. Nanostring is a non-amplification based digital method to quantitate gene expression that uses color-coded molecular barcodes to detect hundreds of transcripts in a sample. Using this approach, we identified unique gene signatures in spleen tissue at days 4, 7, and 12 following WNV infection, which delineated distinct differences between asymptomatic and symptomatic CC lines. We also identified novel immune genes. Data was deposited into the Gene Expression Omnibus under accession GSE86000.

**Table:** 

Specifications	
Organism/cell line/tissue	Mouse, Spleen tissue
Sex	Male
Sequencer or array type	Nanostring pan cancer immune panel
Data format	Raw and normalized matrix provided
Experimental factors	
Experimental features	This analysis identified a subset of novel genetic signatures involved in the resistance and susceptibility of West Nile Virus in Spleen tissue using the Nanostring platform.
Consent	Allowed for reuse. Please contact authors before reuse.
Sample source location	Seattle, WA, USA

## 1 Direct link to deposited data

http://www.ncbi.nlm.nih.gov/geo/query/acc.cgi?acc=GSE86000

## 2 Experimental design

We screened RNA from 95 spleen samples over 8 different CC lines. Across the 8 CC lines, four were defined as symptomatic, the other four were asymptomatic (see Table 1). Samples were captured at 4, 7, and 12 days' post-WNV infection. The nanostring platform uses the ncounter technology, which utilizes 100 nt molecular bar codes (50 nt capture probe and 50 nt reporter probe) to count genes without an amplification step. We used a predestined kit (pan cancer immune) that includes 770 immune related genes.

## 3 Material and methods

### 3.1 Virus

West Nile virus TX-2002-HC (WN-TX) was propagated using previously described methods [4]. Viral stocks were generated using supernatants collected from infected vero cell lines and stored at 80 °C.

### 3.2 Collaborative cross dRIX lines and their disease definitions

The clinical scoring system used to evaluate WNV-infected mice was as follows: 0, healthy mouse (baseline); 1, ruffled fur, lethargy, hunched posture, no paresis, normal gait; 2, altered gait, limited movement in one hind limb; 3, lack of movement, paresis in one or both hind limbs; 4, moribund. Based on weight loss and clinical scoring, CC dRIX (collaborative cross discovery recombinant intercrosses) lines segregated into two broad pathogenic phenotype categories: asymptomatic or symptomatic. Symptomatic was defined as having weight loss > 10% of original pre-infection weight and/or death, whereas asymptomatic was defined as having weight loss < 10% of original pre-infection weight and no death. The CC dRIX lines featured in this study are listed in Table 1.

### 3.3 Mice and infection

CC dRIX lines were bred at the University of North Carolina at Chapel Hill under specific-pathogen-free (SPF) conditions. 6 to 8 week old male mice were transferred to the University of Washington and housed directly in a biosafety level 2 (BSL-2) laboratory within an SPF barrier facility. After a resting period, age- and sex-matched 8- to 10-week old mice were subcutaneously inoculated in the rear footpad with 100 PFU WN-TX. Mice were monitored daily for morbidity (percentage of initial weight loss) and clinical disease scores. Mice were then housed under BSL-3 conditions throughout the experiments, and tissues were processed under BSL-3 conditions. All animal experiments were approved by the University of Washington Institutional Animal Care and Use Committee. The Office of Laboratory Animal Welfare of the National Institutes of Health (NIH) has approved the University of Washington (A3464-01), and this study was carried out in strict compliance with the Public Health Service (PHS) Policy on Humane Care and Use of Laboratory Animals.

### 3.4 RNA extraction and quantitative PCR (qPCR) of WNV

Spleen tissue was removed from mock or WNV infected mice and stored in RNAlater. Tissue samples were homogenized in TRI reagent at 5500 RPM using a Precellys 24 machine. Total RNA was extracted using the Ribopure RNA Purification Kit, with the addition of bromochloropropane. RNA was converted to cDNA using the iScript Select cDNA Synthesis Kit (Biorad). WNV was quantified by probing cDNA with WNV-specific probes through relative expression SYBR Green RT-qPCR, with GAPDH as a loading control.

### 3.5 Nanostring analysis

Nanostring results (raw and normalized counts) were produced from RCC files using nSolver software (version 2.6). Raw comma delimited files will be exported and uploaded to Rstudio (version 0.99.486) with R (version 3.2.4). Over 95% of the genes on the nanostring panel matched to previous matching techniques [8]. Exploratory analysis and summary statistics were calculated to identify variation in the data and relationships among replicates and conditions in each study.

### 3.6 Statistical modeling

We assessed the nanostring data using two statistical approaches. In one approach we normalized the expression data using pre-selected, internal housekeeping genes and plotting subsets of immune-related genes in spotfire (version 7.5.0). In the second approach we performed differential expression analysis across all genes and we filtered raw gene counts (mean ≥ 20) and then normalized the raw counts using the voom Bioconductor package in limma. Linear modeling was then performed in limma using R [1], [6], [7].

### 3.7 Co-expression

Co-expression was performed only on genes that were determined to be statistically significant from the differential expression analysis (threshold: log2 fold change ≥ | 0.58 | and FDR ≤ 0.05) in at least one comparison. Pearson correlations were run on the union of log2FC using the WGCNA and heatmap.2 Bioconductor packages in R [1], [6], [7].

### 3.8 Functional analysis

Ingenuity Pathway Analysis (IPA) and Jepetto (version 1.3.1) were used to determine the biological functions of modules in the co-expression analysis. These tools accept a list of genes and produce a list of known biological functions with an enrichment score corresponding to how significant those genes are to each function [5].

## 4 Conclusion

**Fig. 1:**
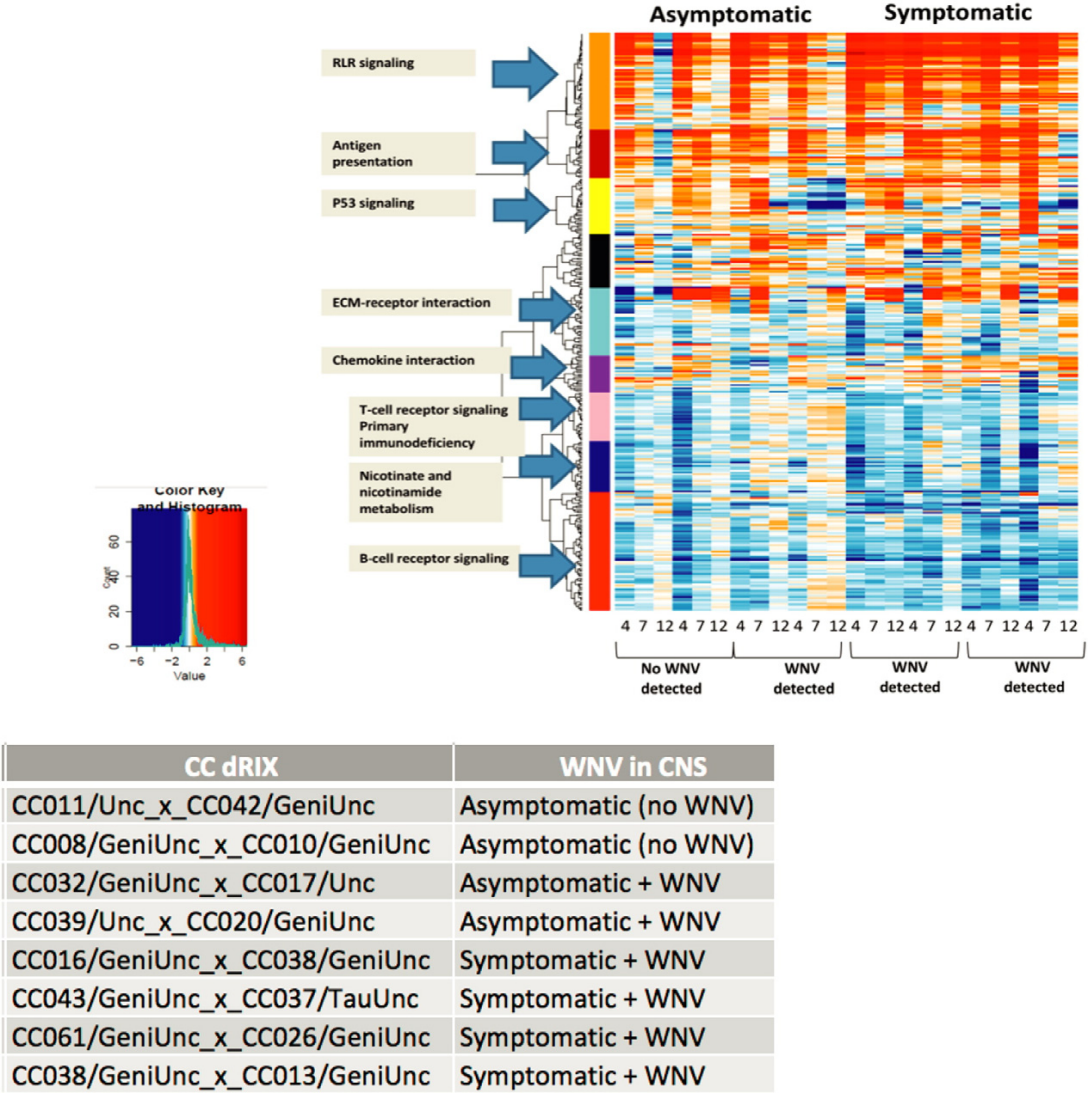
Genomics analysis of WNV-infected spleen tissue from Collaborative Cross mice RNA was extracted from tissue isolated on days 4, 7, and 12 following West Nile virus infection compared to day 2 mock and run on the nanostring immune pan cancer panel. The co-expression data is separated according to WNV disease phenotype (Asymptomatic and Symptomatic) (see methods). The y-axis displays those groups of genes (color modules) that appear co-regulated along with the known biological function. The x-axis displays the number of days post infection.

Differential expression analysis showed hundreds of up-regulated genes in the spleen tissue isolated from both asymptomatic and symptomatic CC dRIX lines. The largest fold change increases in gene expression were detected on day 4 post-WNV infection, especially in the CC dRIX lines defined as asymptomatic to WNV infection (CC039/Unc_x_CC020/GeniUnc, CC008/GeniUnc_x_CC010/GeniUnc, CC011/Unc_x_CC042/GeniUnc), and the induction of these immune genes may be driving protection from WNV disease in these lines. Co-expression analysis showed enriched immune pathways in the spleen tissue isolated from asymptomatic and symptomatic lines. The orange module (Fig. 1) contained genes associated with early immune response including Oas1, Oas3 and Irf7 [9], [10]. The strongest innate response within lines defined as asymptomatic to WNV infection was at days 4 and 7 post-infection, which contrasts with the sustained elevated response observed through day 12 post-infection in the symptomatic lines. We found that the symptomatic lines had slightly more down-regulated genes enriched in B-cell and T-cell receptor signaling. We also discovered expression differences across the genetic backgrounds that share the same response phenotype. Among the asymptomatic lines, we detected differences in co-regulated genes involved in chemokine interactions, P53 signaling, antigen presentation, and the ECM-receptor interaction pathway (Fig. 1).

**Fig. 2:**
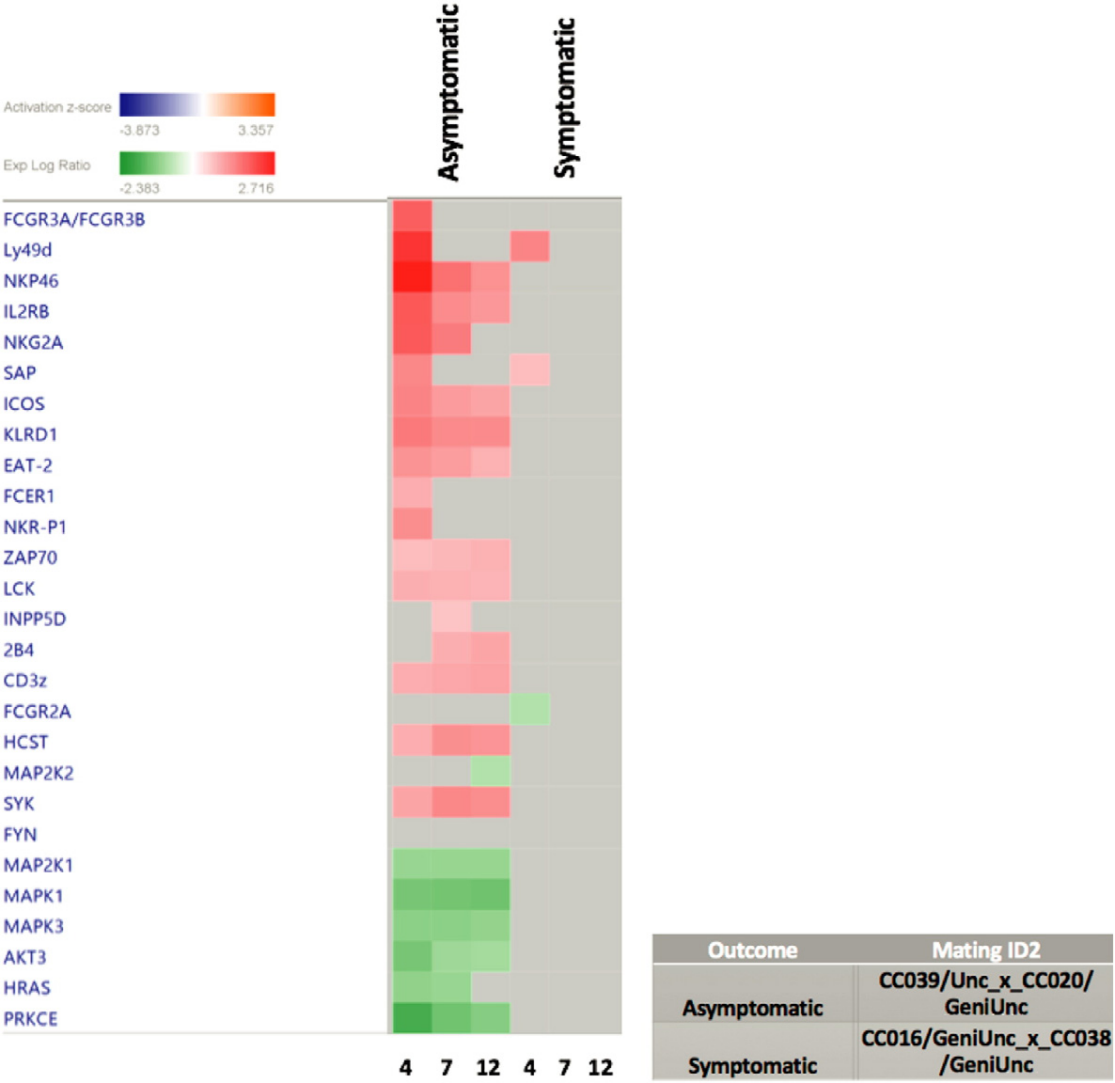
Asymptomatic CC lines have increased expression of genes involved in natural killer cell signaling. Ingenuity Pathway Analysis software (IPA) revealed a clear distinction between an asymptomatic and symptomatic lines and the expression of genes involved in natural killer cell signaling during WNV infection. The Y-axis of the heat map shows the genes associated with natural killer cell activation and the X-axis shows the time points post infection. A comparison of these lines clearly shows a presence versus absence of activation. Those genes marked as red in the table are statistically significant and up-regulated. Those marked as green are also significant and down regulated. Those boxes marked in gray indicate there was gene activity but it did not meet our statistical threshold.

This study represents a step toward capturing the natural variation in immune response within a genetic resource population (Fig. 2) and could provide insight into sources of human variation in responses to WNV infection. The CC is an important biomedical model of human disease and by evaluating this genomic resource using emerging technology we will improve our understanding of host responses that control pathogenic infection.

## Figures and Tables

**Table 1:** Summary collaborative cross tissues evaluated and their associated phenotypic outcome.

CC dRIX	Outcome	Tissue	Timepoints
CC011/Unc_x_CC042/GeniUnc	Asymptomatic	Spleen	D4, D7, D12
CC008/GeniUnc_x_CC010/GeniUnc	Asymptomatic	Spleen	D4, D7, D12
CC032/GeniUnc_x_CC017/Unc	Asymptomatic	Spleen	D4, D7, D12
CC039/Unc_x_CC020/GeniUnc	Asymptomatic	Spleen	D4, D7, D12
CC016/GeniUnc_x_CC038/GeniUnc	Symptomatic	Spleen	D4, D7, D12
CC043/GeniUnc_x_CC037/TauUnc	Symptomatic	Spleen	D4, D7, D12
CC061/GeniUnc_x_CC026/GeniUnc	Symptomatic	Spleen	D4, D7, D12
CC038/GeniUnc_x_CC013/GeniUnc	Symptomatic	Spleen	D4, D7, D12

## References

[1] Loraine A.E., Blakley I.C., Jagadeesan S., Harper J., Miller G., Firon N. (2015). Analysis and visualization of RNA-seq expression data using RStudio, bioconductor, and integrated genome browser. Methods Mol. Biol..

[2] Collaborative Cross Consortium (2012 Feb). The genome architecture of the collaborative cross mouse genetic reference population. Genetics.

[3] Geiss G.K., Bumgarner R.E., Birditt B., Dahl T., Dowidar N., Dunaway D.L., Fell H.P., Ferree S., George R.D., Grogan T., James J.J., Maysuria M., Mitton J.D., Oliveri P., Osborn J.L., Peng T., Ratcliffe A.L., Webster P.J., Davidson E.H., Hood L., Dimitrov K. (2008 Mar). Direct multiplexed measurement of gene expression with color-coded probe pairs. Nat. Biotechnol..

[4] Graham J.B., Thomas S., Swarts J., McMillan A.A., Ferris M.T., Suthar M.S., Treuting P.M., Ireton R., Gale M., Lund J.M. (2015 May 5). Genetic diversity in the collaborative cross model recapitulates human West Nile virus disease outcomes. MBio.

[5] Rasmussen A.L., Okumura A., Ferris M.T., Green R., Feldmann F., Kelly S.M., Scott D.P., Safronetz D., Haddock E., LaCasse R., Thomas M.J., Sova P., Carter V.S., Weiss J.M., Miller D.R., Shaw G.D., Korth M.J., Heise M.T., Baric R.S., de Villena F.P., Feldmann H., Katze M.G. (2014 Nov 21). Host genetic diversity enables Ebola hemorrhagic fever pathogenesis and resistance. Science.

[6] Gentleman R.C., Carey V.J., Bates D.M., Bolstad B., Dettling M., Dudoit S., Ellis B., Gautier L., Ge Y., Gentry J., Hornik K., Hothorn T., Huber W., Iacus S., Irizarry R., Leisch F., Li C., Maechler M., Rossini A.J., Sawitzki G., Smith C., Smyth G., Tierney L., Yang J.Y., Zhang J. (2004). Bioconductor: open software development for computational biology and bioinformatics. Genome Biol..

[7] Smyth G.K. (2004). Linear models and empirical Bayes methods for assessing differential expression in microarray experiments. Stat. Appl. Genet. Mol. Biol..

[8] Oligomask (2014). A framework for assessing and removing the effect of genetic variants on microarray probes. R Journal.

[9] Chakrabarti A., Jha B.K., Silverman R.H. (2011 Jan). New insights into the role of RNase L in innate immunity. J. Interf. Cytokine Res..

[10] Honda K., Yanai H., Negishi H., Asagiri M., Sato M., Mizutani T., Shimada N., Ohba Y., Takaoka A., Yoshida N., Taniguchi T. (2005 Apr 7). IRF-7 is the master regulator of type-I interferon-dependent immune responses. Nature.

